# Older Wheelchair Users Recommend Age-Friendly Design Improvements to a Wheelchair Maintenance App: Mixed Methods Development Study

**DOI:** 10.2196/39301

**Published:** 2022-10-18

**Authors:** Alyssa Boccardi, Fangzheng Wu, Jon Pearlman, Anand Mhatre

**Affiliations:** 1 Department of Rehabilitation Science and Technology University of Pittsburgh Pittsburgh, PA United States; 2 Occupational Therapy Division Ohio State University Wexner Medical Center Columbus, OH United States

**Keywords:** aging, older adults, maintenance, mobile phone, repair, smartphone, wheelchair

## Abstract

**Background:**

Wheelchair part failures have doubled over the past decade. Preventative wheelchair maintenance reduces wheelchair failures and prevents user consequences. We are developing a smartphone app called WheelTrak, which alerts users when maintenance is required, to encourage maintenance practices and compliance.

**Objective:**

This mixed methods study aimed to develop a wheelchair maintenance app using broad stakeholder advice and investigate older adults’ interaction experience with the app and their perceived barriers to and facilitators of maintenance.

**Methods:**

Interviews were conducted with stakeholders, including mobility device users, to generate needs statements and app specifications. The app was designed in 2 stages. Stage 1 involved the development of the app according to the specifications and evaluation of the app interface by lead users. Stage 2 included the revision of the app screens and manual functionality testing. Usability testing and semistructured interviews were conducted with older wheelchair and scooter users. The System Usability Scale was used to measure app usability.

**Results:**

Interviews with power and manual wheelchair users (37/57, 65%), wheelchair service providers (15/57, 26%), manufacturers (2/57, 4%), seating and mobility researchers (1/57, 2%), and insurance plan providers (2/57, 4%) informed the needs and specifications of the app technology. The 2-stage development process delivered a fully functional app that met the design specifications. In total, 12 older adults (mean age 74.2, SD 9.1 years; n=10, 83% women; and n=2, 17% men) participated in the usability testing study. Of the 12 participants, 9 (75%) agreed to use WheelTrak for preventative maintenance. WheelTrak scored an average System Usability Scale score of 60.25 (SD 16). Four overarching themes were identified: WheelTrak app improvements, barriers to maintenance, consequences related to mobility device failure, and smart technology use and acceptance. Older adults preferred the simplicity, readability, personalization, and availability of educational resources in the app. Barriers to maintenance pertained to health issues and lack of maintenance knowledge among older adults. Facilitators of maintenance included notification for maintenance, app connectivity with the service provider, reporting of device failure, and the presence of a caregiver for maintenance.

**Conclusions:**

This study highlighted age-friendly design improvements to the app, making it easy to be used and adopted by older wheelchair users. The WheelTrak app has close to average system usability. Additional usability testing will be conducted following app revision in the future.

## Introduction

### The Calamity of Wheelchair Failures

According to the World Health Organization, older adults will be using ≥2 assistive devices by 2030 to overcome barriers and experience full and equal participation in society [1]. Wheelchairs are assistive devices that serve as a primary means of mobility and independence for older adults and are linked to improved well-being and delayed need for long-term care. Unfortunately, although wheelchairs play a significant role in the lives of older adults and people with locomotor disabilities, they are known to break down frequently. Cross-sectional study findings over the past 2 decades show that approximately 45% to 88% of wheelchair users experience wheelchair part failures every 6 months owing to failures of wheelchair casters, rear wheels, brakes, frames, and seating systems [2-7]. Comparatively, in low- and middle-income countries, where rehabilitation services are scarce and outdoor environments are adverse, part failures occur every 2 to 3 months [8-13]. A high incidence of part failures among older wheelchair users was found in a study conducted in El Salvador [14,15]. Approximately 57% of the older adults have experienced part failures in the past 3 months of wheelchair use owing to high-risk failures of critical wheelchair parts such as casters and wheels. Approximately 75% to 95% of the older participants rated their wheelchairs as unsatisfactory and in unsafe working condition, which can contribute to part failure [14,15]. One-third of wheelchair breakdowns result in adverse events, including injuries, pain, depression, and hospitalization [2,4,6,12,13]. Overall, wheelchair part failures negatively affect the lives of older wheelchair users globally, thus increasing public health and personal burden.

### Need for Wheelchair Maintenance

Community-based and secondary data analysis studies [7,16,17] and the World Health Organization’s guidelines [18] recommend preventative wheelchair maintenance to avoid failures that can lead to breakdowns, which make the wheelchair dysfunctional. A randomized controlled trial with 216 manual wheelchair users found that active checkups and maintenance in 12 months led to no wheelchair accidents in the treatment group [17]. The number of accidents in the control group remained the same. Despite this evidence, preventative maintenance is rarely conducted. This can be attributed to unfavorable health care policies; lack of user training, knowledge, and capability; and lack of tools for repair, among several other reasons. Researchers have developed resources such as training programs and maintenance checklists that include consensus-based, generic maintenance schedules for inspection and cleaning of wheelchair parts to support maintenance practices [17]. However, the checklists cannot monitor wheelchair use in the community and predict wheelchair failures to inform maintenance. In other industries, maintenance schedules are dependent on product use. For instance, in automobiles, the odometer indicates oil change based on the distance traveled by the vehicle [19]. In aircraft and heavy equipment industries, vibration-based condition monitoring systems are used to generate alerts for part replacement and preventative maintenance events and to prevent equipment damage and downtime [20,21]. Unfortunately, no such tools exist to monitor wheelchair use and wear down, determine the probability of high-risk failure, and alert users and wheelchair providers about maintenance and part replacement events.

### Availability of New Technology

The widespread availability of low-cost activity monitoring tools, such as sensors and smartphones, offer an opportunity to track real-time wheelchair use characteristics and guide maintenance. Smartphones are widely available around the world. For instance, in low- and middle-income countries—approximately one-third of older individuals (aged >60 years) and people with disabilities have a smartphone, and that number continues to increase [22-24]. Therefore, we are developing a mobile health (mHealth) technology called WheelTrak to enable use-based maintenance practices for wheelchair users and stakeholders involved in wheelchair repair. The concept comprises a smartphone app connected via Bluetooth to a low-power sensor unit that attaches to the wheelchair and collects road shock data when the wheelchair is in motion. Shocks experienced over time will be benchmarked against a wheelchair wear index (WWI) that can predict the occurrence of critical wheelchair failure. When maintenance is required according to WWI, users can be notified through the WheelTrak smartphone app.

This paper describes the staged design process and usability testing of the WheelTrak smartphone app. The study aimed to evaluate the usability of the WheelTrak app for preventative maintenance and understand the barriers to and facilitators of maintenance, which can inform the future development of the technology. 

## Methods

**Figure 1:**
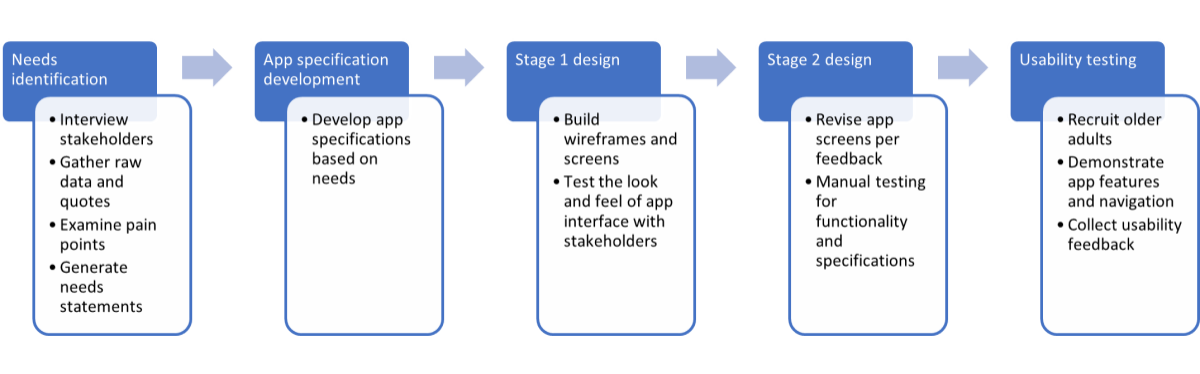
App design and testing process.

A systematic design procedure proposed by Ulrich and Eppinger [25] was used to gather raw data and develop needs statements for the WheelTrak maintenance technology. The design and testing process followed in this study is shown in Figure 1.

### Needs Identification

Multiple stakeholders in the wheelchair industry, who are associated with repairs and maintenance, including wheelchair users, were interviewed as a part of the technology transfer programs at the University of Pittsburgh. The interviews were conducted at a conference, in person at a wheelchair clinic, or via phone. Initial stakeholders were affiliated with the University of Pittsburgh’s Rehabilitation Science and Technology Continuing Education program. Then, stakeholders were recruited using the snowball sampling method or word of mouth at the conference or approached via social media platforms.

Customer discovery style interviews were conducted. Users were asked about their daily life activities and journey as a user of a mobility device. Likes and dislikes about the wheelchair and its use settings were collected. Instances where the user experienced inconveniences or consequences owing to wheelchair part failure or repair were discussed. Furthermore, users were probed regarding their understanding and awareness of preventative maintenance and related training. Opinions on wheelchair failures and repair services from wheelchair providers were collected. Users were asked about the features they would like to see in an mHealth preventative maintenance technology.

Wheelchair service providers were asked about their business operations and challenges, criteria for product selection and prescription, repair experiences of technicians, reimbursement versus costs, cascading effects of repairs on their operations, and experiences with clients and insurance. Providers were queried about the development and integration of new technology in their day-to-day repair-related operations. Wheelchair manufacturers and insurance plan providers were presented generic data on wheelchair part failures and asked to share their viewpoints on the existing state of affairs regarding repairs and maintenance. They were questioned about benefits of and risks with new maintenance-related technology and its integration into service. After each interview, the interviewee was asked for referrals for additional interviews. No identifiable information was collected during the interviews.

Raw data from interviewees were documented using handwritten notes, either by the author (AM) or 2 other researchers (refer to the *Acknowledgments* section). Notes included facts, insights, and quotes stated by the interviewees. Pain points for each stakeholder were extracted from the notes manually. These pain points were considered for generation of needs statements. For the providers, who articulated the needs well, direct quotes were converted into needs statements. Needs statements for the WheelTrak technology (app, sensor, and web-based platform) were generated by the author (AM) and a researcher (refer to the *Acknowledgments* section).

### App Specification Development

On the basis of needs statements related to the maintenance app and benchmarking to existing health apps, functional requirements or specifications were generated for the WheelTrak app by authors (AM and FW) and another researcher (refer to the *Acknowledgments* section). These specifications focused on a low-fidelity version of the app or the minimum viable prototype for usability testing.

### App Design—Stage 1

Using app specifications, screen wireframes were brainstormed by the author (AM) and other researchers (refer to the *Acknowledgments* section) and hand drawn subsequently. These screens were drafted in Adobe Illustrator (Adobe Inc) by a researcher and uploaded to a rapid prototyping platform. The platform established a sequence among the screens. Active, ultralight wheelchair users, who can be considered as lead users, tested the app prototype and provided suggestions.

### App Design—Stage 2

Feedback from the users was implemented by revising the app screens in Adobe Illustrator. Then, the screens were migrated to Android Studio (Google). A low-fidelity WheelTrak app prototype was deployed on a smartphone for manually testing the appearance and functionality of app screens. Following several design iterations performed by the author (FW), the app screens and incorporated features addressed the app specifications.

### Usability Testing

#### Ethics Approval

An institutional review board application (STUDY20100451) was reviewed and approved by the Human Research Protection Office committee at the University of Pittsburgh. Potential participants were contacted via phone, and a script approved by the institutional review board regarding study introduction was followed to seek verbal consent from interested older wheelchair users.

#### Participant Recruitment

Inclusion criteria for participants were as follows: (1) aged ≥60 years and (2) had a manual wheelchair, power wheelchair, or scooter. Participants were recruited through the University of Pittsburgh Pepper Center Registry. Semistructured interviews were conducted with older wheelchair and scooter users to perform usability testing with the WheelTrak app.

#### Usability Testing Procedure

Before the interview was conducted, recruitment, screening, interview availability, and location were determined. We conducted the interviews using a set of questions (Multimedia Appendix 1) for older adults based on early wheelchair user interaction experiences related to WheelTrak development. During the interviews, participants were asked about their experiences with wheelchair failure, repair and maintenance, barriers to and facilitators of maintenance, and technology use. The app designed in stage 2 was downloaded in an Android phone. The interviewer demonstrated selective WheelTrak screens and allowed the participant to use the app afterward. Participants were asked questions regarding their impressions about the app. They were probed regarding whether the app would fit into their lifestyle and how often they would use it. Open-ended questions on the use of app features—wheelchair use data, failure reporting, maintenance notification, and awards—were asked. Finally, participants completed the System Usability Scale (SUS), which is a self-report, validated instrument widely used to assess users’ satisfaction with use, internal beliefs, motivation, attitudes, and intentions toward technology [26].

### Data Analysis

Interviews were audio recorded and then transcribed verbatim. Transcripts were deidentified. A systematic approach to qualitative thematic analysis was used to analyze interview data and identify and develop codes and themes using NVivo (version 12 Plus; QSR International). First, all interviews were read by a researcher (AB) to gain an overview of the content. Open, axial, and selective coding strategies were used, enabling the researchers to interact with, compare, and reduce the data constantly. These strategies create a dynamic and nonlinear process that enables themes to be identified, coded, and interpreted [27]. Open coding is the first level of coding used to identify common concepts and themes expressed through interviews [28]. These ideas were given a descriptive label or code. The second level of coding used was axial coding, which was used to further refine, align, combine, and categorize existing themes that share similar ideas [27]. Finally, selective coding (third level of coding) was used to condense categories identified during axial coding, to discover overarching and main themes from participant interviews [27]. Two researchers (AB and AM) collaborated and created the coded interview results.

To evaluate the usability of WheelTrak quantitatively, the average of SUS total scores of all participants was computed. An SUS score of 80 out of 100 indicates that users are impressed with the app and would recommend it to others. An SUS score of approximately 68 is an average usability rating that indicates scope for improvement, and a score <51 indicates lack of usability and need for improvement [26].

## Results

### Needs Identification Results

Stakeholders listed in Table 1 were interviewed during the Randall Family Big Idea Competition (February 2019 to April 2019) and the School of Health and Rehabilitation Sciences Innovation Challenge (January 2020 to April 2020). Overall, stakeholders appreciated the development of preventative maintenance technology.

**Table 1:** Stakeholder feedback on wheelchair failures, repairs, and maintenance (N=57).

Stakeholder	Participants, n (%)	Pain points	Notable quotes	Needs statements
Power and manual wheelchair users	37 (65)	Failures degrade quality of life Repairs and expensive bills from providers Repair waiting times exceeding 6-7 months Tired of waiting on phone to talk to someone and getting the runaround Never received any wheelchair training Small failures lead to big consequences Need to keep track of wheelchair use Users have to take wheelchair to the provider many times Time off work is common Maintenance is not performed despite training	“The app is totally needed even for smaller repairs on my own chair.” “I could fix my own brakes instead of waiting for a provider.” “You learn to become your own mechanic. It’s easier to do it (repairs) yourself.” “Often the providers have ordered the wrong parts. I have to call providers to remind them to order parts.” “If your chair breaks, you are out of the chair, it is frustrating.” “Maintenance user manual is a piece of paper, nobody sees it.” “Insurance won’t cover loose brakes which can cause falls or tips.”	WheelTrak sends maintenance alerts before device failure. WheelTrak is user-friendly. WheelTrak connects end user to the provider for reporting failures and scheduling repair. WheelTrak allows user to order parts from vendors for small repairs. WheelTrak displays wheelchair use data. WheelTrak is available through a subscription service.
Wheelchair providers	15 (26)	Financial losses from wheelchair repairs	“Repairs are the bane of our existence. This technology can make repairs easy.” “Insurance does not look at long term savings.” “...Seems like they (insurance) purposely drag their feet and stall these things.”	WheelTrak performs repair coordination and scheduling. WheelTrak clubs repairs in a distant area and reduces technician trips.
Wheelchair manufacturer	2 (4)	Loss of reputation	“We will find how our products are performing.”	WheelTrak acts as a wheelchair add-on technology.
Seating and mobility researcher	1 (2)	No immediate consequence	“This will bring peace to the wheelchair sector; suppliers won’t have to go through the hassle of convincing the insurance through paperwork.”	WheelTrak can report health and mobility outcomes.
Insurance plan provider	2 (4)	No consequence, but concerned about the risk to the patient population	“...We can more accurately measure causes for a failure, which is fantastic, and would help with claims justification.”	None reported.

### App Specification Development

The app specifications were generated explicitly in this study for targeted use by wheelchair or mobility device users (Textbox 1).

**App specifications for targeted use by wheelchair or mobility device users.**

- User account setup
  The user should be able to register and log in to the app using an email or their Google or Facebook accounts.
  The user should be able to retrieve and edit their information.
- Communicate wheelchair use data
  The app displays daily and weekly travel distance, speed, and impacts.
- Wheelchair information
  The app collects and displays data on wheelchair purchase, model, and manufacturer.
- Wheelchair failure reporting
  The app records wheelchair failure for communicating repair to the wheelchair provider.
- Push notification
  The app sends a notification when maintenance is required.
- Health data collection
  The app collects mobility outcomes data every 3 months using the validated Functional Mobility Assessment tool [29]. This feature was requested by the sponsors of the study. It was hypothesized that wheelchair condition is related to functional mobility outcomes and health.
- Reward user
  The user scores an award for attaining maintenance milestones.
- Platform and device
  The app can be used on Android phone and tablet.
- Data storage platform
  The app uses Firebase (Google).
- Connectivity
  The app uses Bluetooth Low Energy 4.0.

### Stage 1 App Design Results

**Figure 2:**
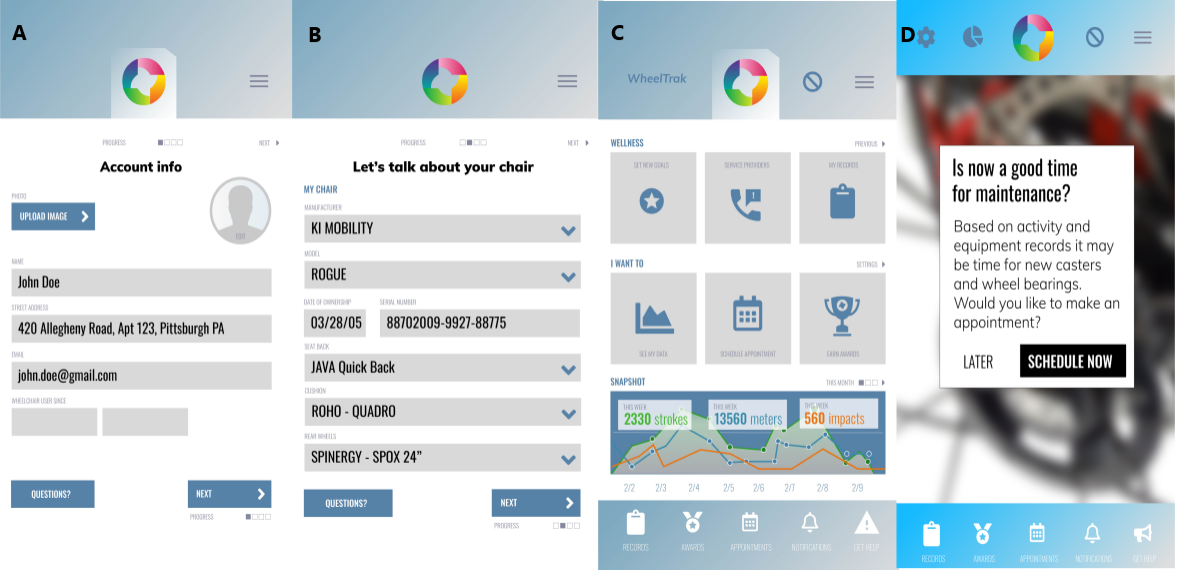
WheelTrak app account setup (A), wheelchair information (B), main screen (C), and maintenance notification (D).

Dummy prototype screens based on specifications and brainstormed wireframes were deployed on Marvel app display engine (Figure 2). In total, 5% (2/37) of the adult wheelchair users navigated through the sequence of screens and were excited at the prospect of having a wheelchair maintenance app for reminders. Both users liked the color contrast, layout, and app features. A user requested for increment in text size and optimization of the display on use factors such as distance, speed, and impacts. Another user requested the inclusion of a parts store, which was beyond the scope of this study.

### Stage 2 App Design Results

**Figure 3:**
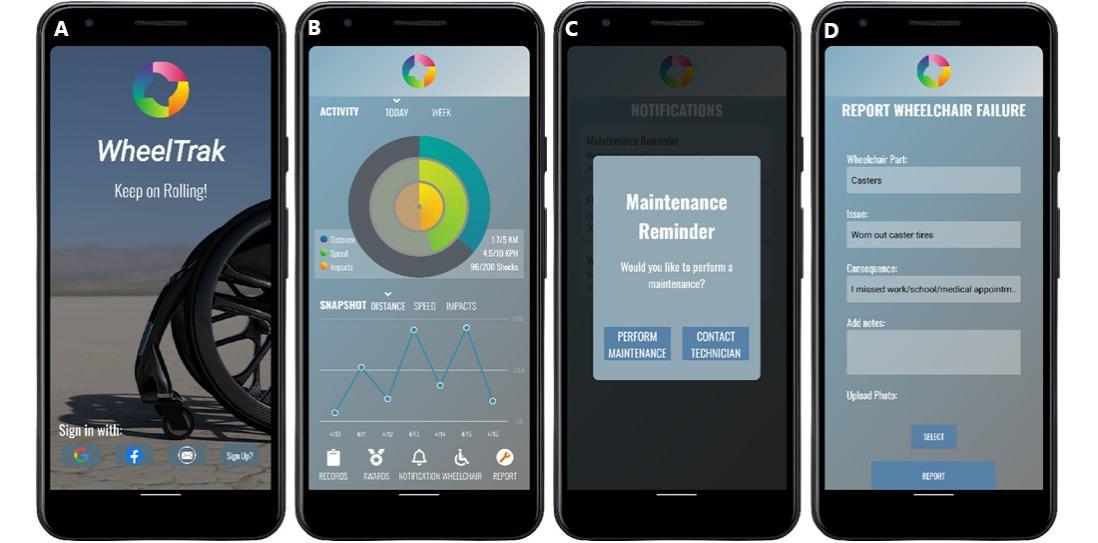
WheelTrak login screen (A), main screen (B), maintenance notification (C), and failure report screen (D).

Apple Health and Fitbit were installed in the phone, and essential health app features, including data display and user account management, were reviewed [30,31]. The feedback from stage 1 and other app reviews informed the development of next version of the screens. The revised screens (Figure 3) were built and deployed on an Android smartphone for iterative specification testing. The app screens and features met the design specifications. The WheelTrak app’s main screen displays wheelchair use data. The *records* tab at the bottom left of the main screen shows daily and weekly use. Wheelchair information collection, failure reporting, health data collection, and scoring of awards are displayed on separate app screens and can be navigated through icons at the bottom of the main screen. User account set up and sensor connection is performed when the user logs in for the first time. Data retrieval from and deposition on the cloud occurs in the background. No glitches or crashes were encountered in the app version used for usability testing.

### Usability Testing Results

In total, 12 older adult wheelchair and scooter users participated in usability testing. Overall, 25% (3/12) of the interviews were conducted via phone and Zoom Meetings (Zoom Video Communications), a web-based meeting platform [32]. The remaining interviews (9/12, 75%) were conducted at the participants’ residence or at the University of Pittsburgh’s Department of Rehabilitation Science and Technology Design Studio. Table 2 presents the demographic characteristics of the participants. Interview themes were classified as shown in Table 3.

**Table 2:** Demographic and wheelchair use characteristics of participants (n=12).

Characteristics			Participants
Age (years), mean (SD)			74.2 (9.1)
**Gender, n (%)**			
	Women	10 (83)	
	Men	2 (17)	
**Type of residence, n (%)**			
	House	5 (42)	
	Apartment	7 (58)	
**Living arrangement, n (%)**			
	Live alone without assistance	2 (17)	
	Live alone with assistance	6 (50)	
	Live with a family member for assistance	4 (33)	
**Mobility devices, n (%)**			
	Manual wheelchair	4 (33)	
	Electric wheelchair	4 (33)	
	Manual and electric wheelchairs	1 (8)	
	Manual wheelchair and walker	1 (8)	
	Scooter	2 (17)	
Stated liking the mobility device, n (%)			12 (100)
**Disliked aspects about wheelchair or scooter, n (%)**			
	Difficulty in avoiding bumping into objects when driving backward	1 (8)	
	Going over thresholds	2 (17)	
	Decreased battery power	1 (8)	
	Wheelchair is uncomfortable owing to spasticity in lower extremities	1 (8)	
	Lack of portability	1 (8)	
**Indoor mobility device activities, n (%)**			
	All indoor activities	4 (33)	
	Some indoor activities (eg, mobility around apartment, house, or job; eating; transfers; mopping floors; and assisting with carrying things from kitchen to dining room)	8 (67)	
**Outdoor mobility device activities, n (%)**			
	All outdoor activities, using vehicle for transportation	3 (25)	
	Activities without vehicle, using own wheelchair	8 (67)	
	Activities just outside the house (eg, sit on the porch)	1 (8)	

**Table 3:** Interview themes (n=12).

Themes and categories				Participants, n (%)
**WheelTrak app improvement**				
	**Improve readability**			
		Increase text size	6 (50)	
		Increase icon size	3 (25)	
		Change pie graph colors	8 (67)	
	**Prioritize simplicity**			
		Simplify	4 (33)	
		Make graphs and charts easy to understand	8 (67)	
		The awards screen of the app is unnecessary for older adults	11 (92)	
	**Include personalization**			
		Use a phone to contact the provider for maintenance, instead of connecting via WheelTrak	4 (33)	
		Send the maintenance notification to the provider through WheelTrak	8 (67)	
		Maintain privacy regarding maintenance events	9 (75)	
	**Education**			
		Provision of manual, guide, or video for maintenance	6 (50)	
**Barriers to maintenance**				
	Lack of maintenance training		11 (92)	
	Low confidence in conducting maintenance		5 (42)	
	Lack of ability to conduct maintenance owing to health issues		8 (67)	
**Consequences related to mobility device failure**				
	Reported failures and repairs		10 (83)	
	Reported consequences after failures		6 (50)	
	Repairs by providers were not timely		3 (25)	
**Smart technology use and acceptance**				
	Use of smartphone or tablet		9 (75)	
	**Stated that they will use WheelTrak app**		9 (75)	
		Only when they remember	1 (8)	
		Once a day	1 (8)	
		Once a week	1 (8)	
		If they had a bumpy ride or travel	1 (8)	
		When they received a maintenance notification	1 (8)	
		Everyday	4 (33)	

### Comments and Additional Findings on WheelTrak App Improvement

Comprehension of the information displayed on the app screens varied across participants and depended on their health conditions and technology use experiences:

I’ve had a cataract operation, so it’s hard to read.

Participant 5

The text size is too condensed it would be more beneficial to have a font that everyone can read.

Participant 4

In addition, 67% (8/12) of the participants reported difficulty in reading the chart, especially the pie graph owing to the similarity in colors. The size of icons at the base of the main WheelTrak screen created complications in navigating through various app screens. Participant 3 does not have a smartphone, so it was difficult for them to touch the icons and input information. Participant 10 and participant 6 experienced seizures and difficulty in touching the icons.

Simplification of the app was noted as a priority by 33% (4/12) of the participants:

...If you want old people to use the app then you have to make it simpler.

Participant 3

The 33% (4/12) of the participants indicated improvements to the information and graphical elements on the main screen.

WheelTrak app screens for failure reporting, wheelchair information collection, and notifications were considered to be the most important by the participants. Convenience was a common feature among these pages owing to access to provider information. One-third of the participants (4/12, 33%) valued the connection with a wheelchair provider for maintenance and repair purposes. Half of the study participants (6/12, 50%) liked the functionality of reporting wheelchair failures using the app. By contrast, most participants (11/12, 92%) reported the awards page to be unnecessary and noted that they would not be motivated by it. Overall, 50% (6/12) of the participants indicated that it would be helpful to have a manual, guide, or training video for learning wheelchair parts, app functions, and maintenance tasks.

When asked how participants would like to be notified about maintenance, phone call, SMS text message, or email were the preferred choices. Overall, 75% (9/12) of the participants preferred privacy regarding maintenance notification. They feared that if their children or spouses were notified of maintenance, it will increase their care burden.

### Comments on Barriers to Maintenance

In total, 92% (11/12) of the participants in the study were not trained in mobility device maintenance. A collective lack of maintenance knowledge and confidence was observed:

You don’t want to mess anything up and just like me, I don’t know how to really do the parts, so I would have to learn about it first.

Participant 6

Additional barriers noted were health issues, including vision problems and lack of strength and dexterity in the hands, making manipulating objects difficult. Of the 12 participants, 3 (25%) stated that they would ask someone to do maintenance. Approximately one-third of the study participants (4/12, 33%) conducted maintenance activities including fixing armrests, inflating tires, and replacing scooter spark plug. Participant 11 received training from a wheelchair maintenance program and conducted maintenance with assistance from their spouse.

### Comments on Consequences Related to Mobility Device Failure

Overall, 83% (10/12) of the participants reported wheelchair repairs and failures before the study. Brakes, wheels, and tires incurred the most failures. These parts were replaced during repair. Other failures were found with armrests, battery connections, grip handles, back support, front struts, and cushions. In total, 50% (6/12) of the participants reported consequences associated with these failures. Participant 3 and participant 10 stated that they are aware that their brakes are not secure and may slip, causing them to be more cautious and fearful when standing up. The scooter of participant 2 broke down on a transportation bus and they could not perform their daily life activities afterward. Overall, 17% (2/12) of the participants reported receiving a loaner chair that they did not like, thus causing frustration. Most repairs were completed by wheelchair service providers or the Veteran Affairs. They were timely, except for 25% (3/12) of the participants. Participant 6 stated that their repair took a week, whereas participant 7 stated that it took 2 months. Participant 11 experienced a front caster fracture failure during regular use and stated that their wheelchair has not been repaired yet. Overall, >4 months have elapsed, and the participant still uses a loaner chair.

### Comments on Smart Technology Use and Acceptance

More than half of the older wheelchair user participants (9/12, 75%) used smart technology, that is, smartphones and tablets. Participants expressed proficiency in using their flip phones, home phones, or smartphones. All participants (12/12, 100%) indicated that they keep their phones on them or close to them. These places include their bag, wheelchair pocket, pants pocket, rollator basket, and kitchen or bedroom. Overall, 17% (2/12) of the participants indicated that they kept their phones on them to report falls. Regarding usability, the most significant barrier noted was remembering to charge the devices. Almost all participants (11/12, 92%) noted that they charge their phone daily or when it alerts them about low battery. The participants disliked short charging cords or plugging the charging cable into the wall socket.

### Additional Feedback on WheelTrak Technology

All study participants (12/12, 100%) recognized the importance of routine maintenance for wheelchairs during the interviews. In total, 75% (9/12) of the participants appreciated WheelTrak’s development and wanted to use the technology (app and sensor). Of the 12 participants, 3 (25%) participants (participant 6, participant 8, and participant 11) did not favor using the maintenance technology. Participant 6 reported that her wheelchair has electronics that track daily distance and speed:

I know my chair pretty well when something is going to break, so I don’t need the app.

Participant 6

Participant 8 and participant 11 reported that they would not use the app because they are inactive. Participant 6 and participant 8 experienced wheelchair failures and subsequent consequences before the study.

### Quantifying WheelTrak Usability

**Figure 4:**
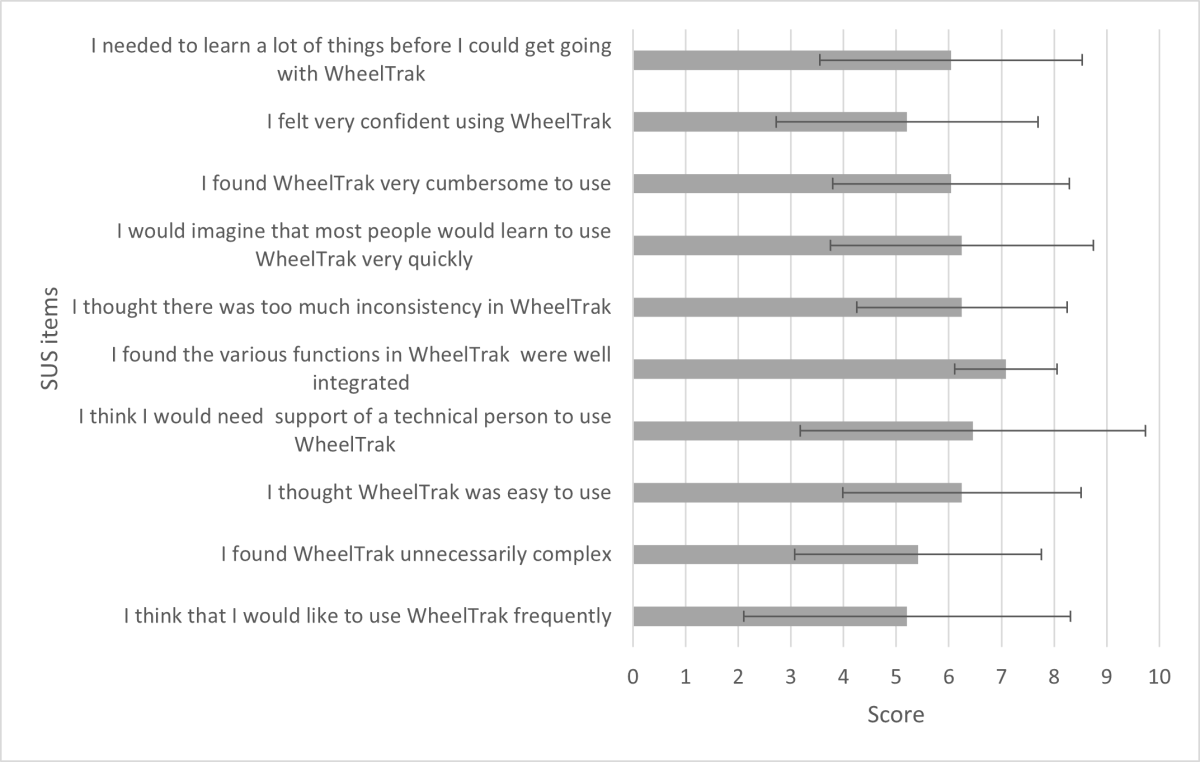
Mean scores for individual SUS items.

The average SUS score was 60.25 (SD 16). Figure 4 shows the average score for each SUS item. As far as individual scores are concerned, participant 6 had the highest SUS score of 90 but stated that they would not use WheelTrak. They expressed that the app was designed well and easy to use. Participant 1 had the lowest SUS score of 35; they were unfamiliar with using a smartphone and experienced extreme difficulty in using the WheelTrak app.

## Discussion

### Principal Findings

Our study demonstrates that older adults using wheelchairs favor the use of the WheelTrak smartphone app for conducting preventative maintenance and suggests age-friendly modifications to improve the usability of the app.

Preventative maintenance reduces the frequency of wheelchair failures and breakdowns, thus preventing adverse health consequences to older users [7,16,17]. To encourage maintenance practices and compliance, we are leveraging the existing capabilities of smartphones and sensors and developing an mHealth app called WheelTrak. A systematic design process used to gather technology design requirements revealed multiple development areas for maintenance technology, including the app, from the perspective of diverse stakeholders. This study specifically aimed to evaluate the usability of and barriers to the WheelTrak smartphone app with the older subset of the target user population. All the older wheelchair and scooter user participants in the study (12/12, 100%) recognized the importance of conducting maintenance, and 75% (9/12) of them expressed interest in using WheelTrak for maintenance purposes. This demonstrates that although some older adults did not use smartphones or tablets, they still perceived WheelTrak positively. In addition, participants rated the WheelTrak app for close to average usability, according to the SUS score. They recommended design improvements to make WheelTrak more inclusive for use and adoption by the older population.

Facilitators for app-based preventative maintenance included smart technology use, relevant app features, and interest in conducting maintenance with support from family member. Overall, 75% (9/12) of the participants used smart technology, which is slightly more than the statistics reported by the Pew Research Center [33]. Most smart technology users (9/12, 75%) were interested in using the WheelTrak app for maintenance. Specifically, the features such as maintenance reminders, failure reporting using photos of failure, and ability to connect with the provider were much appreciated. This outcome indicates that the WheelTrak technology provides convenience and relief from pain points on failures and scheduling repairs, as listed in Table 1. These pain points have plagued the wheelchair user community for decades. Users were motivated to leverage the existing resources and family member or caregiver support for conducting maintenance. Aligning with the notable quotes listed in Table 1, users are interested in conducting small repairs, which can avert the occurrence of major failure-related consequences.

As measured by the SUS instrument, the usability of the WheelTrak app exhibits significant variability across the SUS items, as seen in Figure 4. Key reasons for such variability can be attributed to familiarity with and challenges in using digital technology, confidence in device maintenance, usability issues with the current WheelTrak app version, and existing bias toward mobility device capabilities, as found during the study. These reasons may be characteristic of the older population of wheelchair users. These app usability findings prompt the development of a new app version for older adults, based on feedback collected in this study.

The WheelTrak app has close to average usability rating (SUS score=60.5), as the app features are yet to be tailored to the capabilities of older adults. When app screens were demonstrated to older users, comprehension of wheelchair use information through animated line graphs was difficult. Challenges were encountered with navigation through different screens. Although all the participants (12/12, 100%) conceded the importance of maintenance, most of them (8/12, 67%) experienced health limitations to perform general maintenance. Such limitations are commonly noted as barriers in mHealth literature about older adults. Limited physical ability, cognition, perception, and motivation have been cited as barriers to using mHealth technologies [34]. In addition, as highlighted in a previous app review study [35], the variations in capabilities of our study cohort showed that one size does not fit all; hence, we need to tailor the app to different older adult personas. We plan to address the barriers found during usability testing and accommodate the recommendations suggested by the older adults.

Older adults recommended simplicity in visualization and information communication. Accordingly, we plan to develop new specifications for text and icon size. The amount of information on each page shall be reduced and displayed in text format for easy comprehension. We may use paper prototyping and testing to develop such specifications and test new app workflows. Use graphs, color fading, and animations can be removed completely. Similarly, the awards and records screens are not appealing to the older adults and can be removed. The valuable feedback collected in this study on app design improvements—optimal typography, color contrast, icon size, and information personalization—can apply to other apps developed for the older population of wheelchair users.

Personalization of app features is highly valued by older adults. The users want to exercise control over app communication. Privacy of use and maintenance data and communication of maintenance events via phone or app will be based on user selection. These options could be selected during the installation of the app, with support from a technical person who may be a family member, caregiver, assistive technology professional, or wheelchair provider technician.

All except 1 participant (11/12, 92%) were unaware of maintenance training and lacked technical knowledge about wheelchairs. This finding was synonymous with the user feedback collected during needs assessment. Although barriers to training availability exist, half of the study participants (6/12, 50%) expressed willingness to educate themselves about maintenance through videos, manuals, and resources in the app. The WheelTrak app can incorporate such training materials to increase maintenance knowledge, familiarize users with WheelTrak app–enabled maintenance, and assist users in taking charge of maintenance. Furthermore, we anticipate the integration of WheelTrak technology in the in-person and remote wheelchair maintenance training programs to augment maintenance training capabilities and outcomes [36]. With increasing smartphone adoption among older adults and people with disabilities, a low-cost tool such as WheelTrak can assist in scaling maintenance training and enable great compliance.

Most participants (8/12, 67%) expected WheelTrak to alert providers about upcoming maintenance events. This motivates us to explore the development of WheelTrak for the wheelchair provider group. Providers incur wheelchair repair losses, a finding from provider feedback during needs assessment and a cross-sectional study that surveyed >125 providers [37]. According to the provider’s feedback obtained during the app design phase, these losses can be prevented if WheelTrak can streamline and schedule upcoming repairs. Accordingly, web-based platform development can be investigated in the future for providers.

Participants’ experiences with wheelchair failures and repairs are similar to those reported in the literature [3-5] and needs assessment phase. These events result in long time without the wheelchair and severely limited mobility, as repair wait times stretch beyond months, which means spending more time in bed [3]. It is not surprising that wheelchair failures are associated with pressure injuries and rehospitalization [6]. Consequences include time off work, numerous calls and trips to the provider, and expensive repair bills if insurance does not cover repair. In addition, older users’ frustrations with loaner wheelchairs were documented in this study.

A trained participant (participant 11) who conducts maintenance experienced a high-risk failure during regular wheelchair use. These findings support the development of WheelTrak technology for monitoring wheelchair condition and informing users about upcoming high-risk failures and maintenance events. WheelTrak plans to predict failures, especially those related to wheels and brakes. These parts need to be replaced to avoid risks related to tips and falling out of the wheelchair, which can cause injury to the user.

The barriers and consequences realized in this study and recent studies on repair experiences of wheelchair providers demonstrate that it is crucial to address the repair-related needs of mobility device users and providers. Furthermore, as WheelTrak develops, health care policies must enact provisions and support preventative maintenance practices. Reimbursements for service and maintenance activities can be allowed with justification provided by ground truth data collected by WheelTrak.

### Limitations

First, the design phase cast a wide net for gathering WheelTrak app requirements, but the usability test was conducted only with older adults, a subset of the intended WheelTrak user population. This may have affected the SUS usability score. Second, the usability testing study concentrated on app navigation and interaction and was limited to specific app screens that older participants would use. For instance, user account setup, sensor connectivity via Bluetooth, cloud connectivity, and health information collection using the Functional Mobility Assessment tool are features available in the app. These features will be tested in future studies with other user cohorts. Finally, we aimed to limit potential bias in soliciting positive feedback for WheelTrak. For this purpose, AB was initially recruited to conduct interviews.

### Future Studies

We plan to develop an age-friendly version of the app based on study results and conduct focus group testing. In addition to SUS, we plan to include other validated tools to understand users’ cognitive load while performing tasks in the app. As part of ongoing studies, the app’s current version will be tested with other mobility-assistive device users (aged 60 years) to identify their perceptions and usability of WheelTrak. WheelTrak development will include other modes of informing participants about maintenance such as SMS text messages, email, and phone calls, in addition to app notifications. Along with app development, we are conducting field studies to monitor wheelchair use and develop a WWI-based preventative maintenance model.

### Conclusions

The WheelTrak preventative maintenance app has been identified as a tool that older adults can use for maintenance notifications and reporting wheelchair failures to providers. Despite challenges in using smart technology, older adults expressed interest in educating themselves about maintenance and conducting WheelTrak-led maintenance with caregiver or provider support. The WheelTrak app has close to average usability for older adults with disabilities. Findings from the study informed the research team about improvements to the app, making it easy to be used and adopted by wheelchair users across their life span.

## Appendix

Multimedia Appendix 1 Interview guide.

## Abbreviations

mHealth: mobile health
SUS: System Usability Scale
WWI: wheelchair wear index

## Data Availability

The data sets generated and analyzed during this study are available from the corresponding author upon reasonable request.
