# Supplementary material for: Older Wheelchair Users Recommend Age-Friendly Design Improvements to a Wheelchair Maintenance App: Mixed Methods Development Study
Source: JMIR Aging. 2022 Oct 18;5(4):e39301. doi: 10.2196/39301 (PMC9627462; doi:10.2196/39301)
Supplement: Multimedia Appendix 1 [file aging_v5i4e39301_app1.docx]

Appendix 1

**Interview Guide**

## Opening Questions

Hi, thank you for taking time today to talk with me. Today I will be gathering your thoughts on wheelchair performance and maintenance and feedback on a smartphone application called WheelTrak for maintenance, but before I give you more information about the app, I want to get to know you a little more.

1. What do you like to do in your free time?
2. What activities do you use your wheelchair for indoors?
3. What activities do you use your wheelchair for outdoors?
   1. Do you use your wheelchair for travel (to work or community center)?
4. Do you need assistance performing any daily activities?
   1. If so, what are these activities?
5. Do you live alone, or do you have someone at home with you?
6. How do you like your wheelchair?
   1. Does it perform as per your expectations?
      1. List examples where it does meet or not meet your expectations?

## Breakdown, Repairs, Maintenance

1. Describe your experience related to wheelchair failure.
   1. What broke?
   2. What breaks more often?
   3. Any consequences or adverse effects due to failure?
      1. Injuries?
      2. Stranded in the street?
      3. Did you miss out on work and/or loss of pay?
      4. Could you meet with family and friends?
2. Describe your experience with repairing your wheelchair.
   1. What components needed repairs?
   2. Who completed the repair?
   3. How long did this process take?
   4. Describe your experience (if any) with service providers.
      1. Have you received bills for repairs?
      2. Have you paid them?
      3. If no,
         1. What happens if you do not?

## Barriers/Facilitators

Maintenance may include periodic repairs and replacement of wheelchair parts.

1. Have you received training in wheelchair maintenance?
2. Do you currently perform wheelchair maintenance?
   1. If yes:
      1. What parts do you perform maintenance on?
      2. Do you need assistance?
      3. Do you have the tools?
      4. What is your personal knowledge on wheelchair maintenance?
      5. How long have you been completing maintenance on your wheelchair?
   2. If no:
      1. What restricts you from doing wheelchair maintenance?
      2. Have you ever considered doing this? Why or why not?
      3. Have you ever performed wheelchair maintenance in the past?
         1. If yes, why did you stop?
      4. What are some ways that can help you engage in wheelchair maintenance?
3. What are the additional challenges to wheelchair maintenance?

## General Technology Questions

1. What are some of the common technologies you use? (Smartphone, home phone, tablet, electric toothbrush, razor, etc.)
   1. Where do you keep your phone on you?
   2. What type of a charger do you use for these devices? (iPhone/micro USB plug, stand charger base, wireless charger)?
      1. Do you charge the device on a daily basis?
      2. Do you have any challenges using the charger?
      3. Is there anything you can think of that would make the charging process easier?
      4. Do you need assistance from someone to charge or operate the device?
   3. What are some challenges with using this technology?

## WheelTrak Use

Now we will be discussing and using the technology called WheelTrak. It has a sensor that goes on your wheelchair and an app the connects with the sensor and displays wheelchair travel distance, speed, shocks, and vibrations to determine when maintenance is due by notifying the user and their caregiver on the app. I will now demonstrate the app.

*App Demo*

- You can sign up/in with google, Facebook, or an email address
- Main page: Keep track of your daily/weekly wheelchair activities via the digital chart
  - Blue = distance
  - Green = speed
  - Orange = impacts
- There are five other pages, let’s look at these:

1. Records page- find out more details about activities in the past 24hours, it shows the time and daily activity for week
2. Awards page – keep you motivative in exercising and maintaining wheelchair. Unfinished achievements are in grey. Finished achievements are in color. Leaderboard enables you to see top users who have traveled longest distances. You can see where you are ranked.
3. Notification – Answering questions on this page will help us calibrate our predictive algorithm. You will receive a reminder on this page if we detect that your wheelchair needs maintenance.
4. Wheelchair page – input your wheelchair info, you can then can report wheelchair failure or contact service provider if needed
5. Report Failure page – you can seek help via reporting about your wheelchair failure (wheelchair part, issue, consequence, upload photo)

Would like to try the app?

(Ask the following questions after the user has navigated the app for 2-3 minutes)

- Can you locate the main page?
- Can you show me how you would input wheelchair info on the app?
- How would you report a failure?
- Can you navigate to the notifications page where upcoming and due maintenance tasks would be?

## App Feedback

1. What are your first impressions of the app?
   1. What did you like or dislike about using the app?
2. What do you think about the design layout of the app?
   1. What did you think about the text/icon size?
   2. Do you have a preference of what type of look you would like the preventative maintenance app to have? If so, what would that be?
3. Was it easy or difficult for you to use the app?
   1. How did you feel about navigating to different pages in the app?
   2. How did you feel about answering questions or inputting information into the app?
4. What do you think about the reporting a failure feature (wheelchair part, issue, consequence, upload photo, contact service provider)?
5. What do you think about the main page daily/weekly wheelchair activities (distance, speed, impacts)?
   1. How did you feel about the charts on this page?
6. What do you think about the records page (activity detail past 24 hours)?
7. What do you think about the Awards page (achievements, leaderboard)?
8. What do you think about the Wheelchair page where you input information about your wheelchair so you can report failure or contact a service provider?
9. What were your impressions on the notifications page (reminders about maintenance)?
   1. Who would you like to be notified when maintenance is upcoming or due?
   2. In addition to notifications displaying in the app, how else would you like to be notified of wheelchair maintenance? (Ex: text message on a cell phone, phone call etc.)
   3. Would you like the app or the sensor to display/sound an alarm on when maintenance is upcoming or due?
   4. Would you like an option on the app that disables the maintenance notification to someone?
      1. Would you want others to have access to this information also? (Answer yes or no to the following):
         1. Family
         2. Friends
         3. Insurance Company
         4. Wheelchair Manufacture
         5. Service Provider
10. How do you feel about this app fitting in with your lifestyle?
11. What would make this app easier to follow or understand?
12. How often would you be willing to use the app?
    1. Would you use it a few hours per day? Only when you remember to? The whole day?
13. What type of charger would you prefer? Wired or wireless?
14. Would you like to charge the WheelTrak sensor regularly?
15. What other information do you wish this app can provide you?
16. Do you have additional comments on wheelchair maintenance using a technology?
17. Any comments for us?
